# Structure and flexibility of the DNA polymerase holoenzyme of vaccinia virus

**DOI:** 10.1371/journal.ppat.1011652

**Published:** 2024-05-20

**Authors:** Wim P. Burmeister, Laetitia Boutin, Aurelia C. Balestra, Henri Gröger, Allison Ballandras-Colas, Stephanie Hutin, Christian Kraft, Clemens Grimm, Bettina Böttcher, Utz Fischer, Nicolas Tarbouriech, Frédéric Iseni

**Affiliations:** 1 Institut de Biologie Structurale (IBS), Université Grenoble Alpes (UGA), Commissariat à l’Energie Atomique et aux Energies Alternatives (CEA), Centre National de la Recherche Scientifique (CNRS), Grenoble, France; 2 Institut de Recherche Biomédicale des Armées, Brétigny-sur-Orge, France; 3 Biozentrum, Universität Würzburg, Würzburg, Germany; Medical University of South Carolina, UNITED STATES

## Abstract

The year 2022 was marked by the mpox outbreak caused by the human monkeypox virus (MPXV), which is approximately 98% identical to the vaccinia virus (VACV) at the sequence level with regard to the proteins involved in DNA replication. We present the production in the baculovirus-insect cell system of the VACV DNA polymerase holoenzyme, which consists of the E9 polymerase in combination with its co-factor, the A20-D4 heterodimer. This led to the 3.8 Å cryo-electron microscopy (cryo-EM) structure of the DNA-free form of the holoenzyme. The model of the holoenzyme was constructed from high-resolution structures of the components of the complex and the A20 structure predicted by AlphaFold 2. The structures do not change in the context of the holoenzyme compared to the previously determined crystal and NMR structures, but the E9 thumb domain became disordered. The E9-A20-D4 structure shows the same compact arrangement with D4 folded back on E9 as observed for the recently solved MPXV holoenzyme structures in the presence and the absence of bound DNA. A conserved interface between E9 and D4 is formed by a cluster of hydrophobic residues. Small-angle X-ray scattering data show that other, more open conformations of E9-A20-D4 without the E9-D4 contact exist in solution using the flexibility of two hinge regions in A20. Biolayer interferometry (BLI) showed that the E9-D4 interaction is indeed weak and transient in the absence of DNA although it is very important, as it has not been possible to obtain viable viruses carrying mutations of key residues within the E9-D4 interface.

## Author summary

The 2022 outbreak of mpox is caused by monkeypox virus closely related to the best-studied model, vaccinia virus. Genome replication, which takes place largely autonomously in the cytosol of the infected cell, is still not really understood. Viral DNA synthesis involves a DNA repair enzyme, the uracil-DNA glycosylase D4 linked to the structural protein A20 forming the processivity factor, which in turn binds to the DNA polymerase E9 forming the complex required for processive DNA synthesis. Here we present the 3.8 Å structure of the apo form of vaccinia virus polymerase holoenzyme E9-A20-D4 obtained by cryo-electron microscopy. Several recent structures from monkeypox virus, with or without bound DNA, and our structure provide static views of the complex revealing a previously undescribed contact between E9 and D4. Our small-angle X-ray scattering data show that other, more open conformations without the E9-D4 contact exist in solution using the flexibility of two hinge regions in A20. Based on site-directed mutagenesis and binding studies, we show that the contact between E9 and D4, which serves to encircle the template strand for processive DNA synthesis, is essential, although the interaction is weak and transient in the absence of DNA.

## Introduction

With the 2022 epidemic outbreak of mpox caused by human monkeypox virus (MPXV) of clade IIb, poxviruses are again in the headlines. Previous human monkeypox infections mainly remained localized in West and Central Africa, where viruses from clades I and IIa circulate in rodent reservoirs [1]. In humans, the two clades lead to zoonotic infections with different degrees of severity. In the past, sporadic infections occurred outside Africa and have always been transmitted by pet rodents. For the first time, the 2022 outbreak involved human-to-human transmission outside Africa leading to an acceleration of the evolution of the viral genome [2]. Human monkeypox virus is mainly transmitted by sexual contacts between men with characteristic skin lesions in the oral and ano-genital area, fever and myalgia, which could be followed by centrifugal secondary eruptions [3]. Available antivirals are tecovirimat [4] interfering with virion assembly and brincidofovir, which targets viral DNA replication through an action as chain terminator (see [5] for a review). MPXV is closely related to vaccinia virus (VACV), the best-studied orthopoxvirus, initially used for smallpox eradication by vaccination. It replicates and assembles in perinuclear viral factories where viral DNA synthesis takes place independently of the host cell nucleus [6]. To date, numerous aspects of the replication cycle remain unsolved. The VACV genome is a 196 kbp double-stranded DNA circularized at the extremities with peculiar telomere structures with imperfect base pairing preceded by repeated sequences [6]. It has been proposed that an origin of replication is located within the telomere [7]. The replication mechanism is still controversial and proceeds most likely through a variant of a rolling circle mechanism suggested by the presence of a primase activity [8] and Okazaki fragments [9]. In addition to the polymerase holoenzyme, the helicase-primase D5, the single-stranded DNA binding protein I3 and the phosphoprotein H5 [10] are required for DNA replication [6]. H5 forms tetramers, binds to DNA and RNA [11] and is supposed to form a hub for other interactors [11]. Roles in RNA processing, viral morphogenesis [12] and very recently as processivity factor of the DNA polymerase [13] have been shown. There has been a long quest for the structure of the poxvirus DNA polymerase holoenzyme since it has been shown that processive DNA synthesis by VACV E9 requires a cofactor composed of VACV A20 and the viral uracil-DNA glycosylase (UNG), D4 [14,15]. The polymerase E9 is a member of the DNA polymerase family B possessing DNA polymerase and 3’-5’ proofreading exonuclease activities [16]. Interestingly, it was also shown to catalyze annealing of single-stranded DNA [17], an activity not found in other family B DNA polymerases. The end-joining reaction requires the 3’-5’ exonuclease activity of E9 that degrades the extremities of dsDNA to create 5’-ssDNA overhangs. E9 on its own was shown to be distributive under physiological conditions, adding only few nucleotides per binding event [18]. An initial low-resolution model of the complex [19] has been completed progressively by high-resolution structural information on several proteins and subcomplexes [20–22]. Still, the mechanism of the processivity factor remained largely unknown. Sparked by the mpox outbreak, interest switched from VACV to MPXV where the proteins of the DNA replication machinery are about 98% identical at the amino acid sequence level. The VACV nomenclature of the reading frames is used throughout the article although the E9-A20-D4 complex is named F8-A22-E4 for MPXV. A first high-resolution structure of the polymerase holoenzyme with bound template DNA, primer strand and incoming dTTP nucleotide obtained by cryo-electron microscopy (cryo-EM) was published by Peng and coworkers [23]. It showed a previously unidentified interface between E9 and D4 allowing the E9-A20-D4 complex to encircle the template strand leading to an unexpectedly compact structure of the holoenzyme. In this model, A20 appears to play a role of a connector and the active site of D4 with its DNA-binding capacity is not used. Further structures of the MPXV polymerase holoenzyme [24,25] showed a DNA-free state with a similar circular structure and an E9-D4 contact, but also a dimer of trimers [24]. Very recently, structures of the MPXV holoenzyme-DNA complex including a H5 tetramer acting as an additional processivity factor connecting A20 to the neosynthesized dsDNA (at a position similar to the one of the PCNA processivity factor of family B DNA polymerases) have been published [13,26].

Here, we present a system for the production of the VACV E9-A20-D4 holoenzyme and its 3.8 Å structure obtained by single particle cryo-EM. As the model disagrees with data obtained in solution by small-angle X-ray scattering (SAXS), we predict the existence of open forms of the complex. We further study the importance of the E9-D4 contact in the absence of DNA and put the VACV polymerase holoenzyme structure in the context of recently determined MPXV structures.

## Results

**Fig 1:**
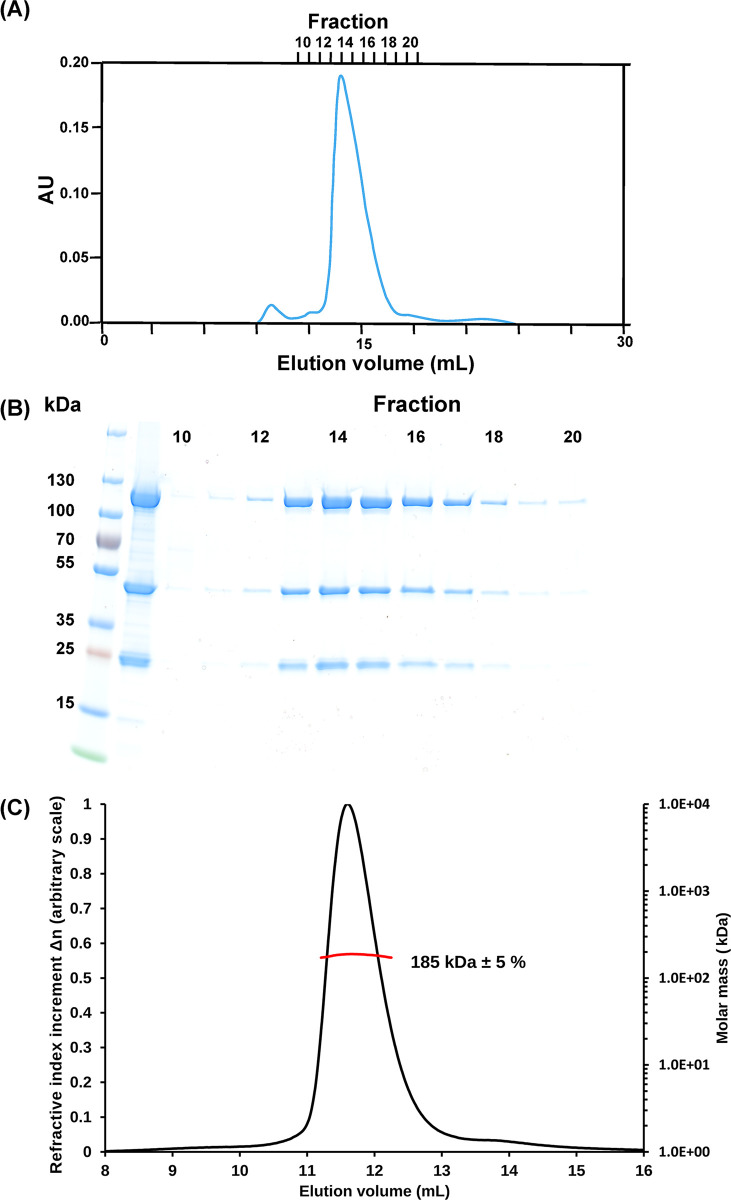
Production of the VACV E9-A20-D4 complex. (**A**) Chromatogram of the final size exclusion step of the purification. (**B**) Fractions of panel (A) analyzed by SDS PAGE. The loaded sample is shown between marker proteins and fraction 10. (**C**) Result of a SEC-MALS run of the complex concentrated from fractions 13 to 17 in panel (B). The red line shows the molecular weight of the complex calculated from the measured scattering signal and the protein concentration determined from the refractive index increment Δn (continuous black line) using dn/dC.

**Fig 2:**
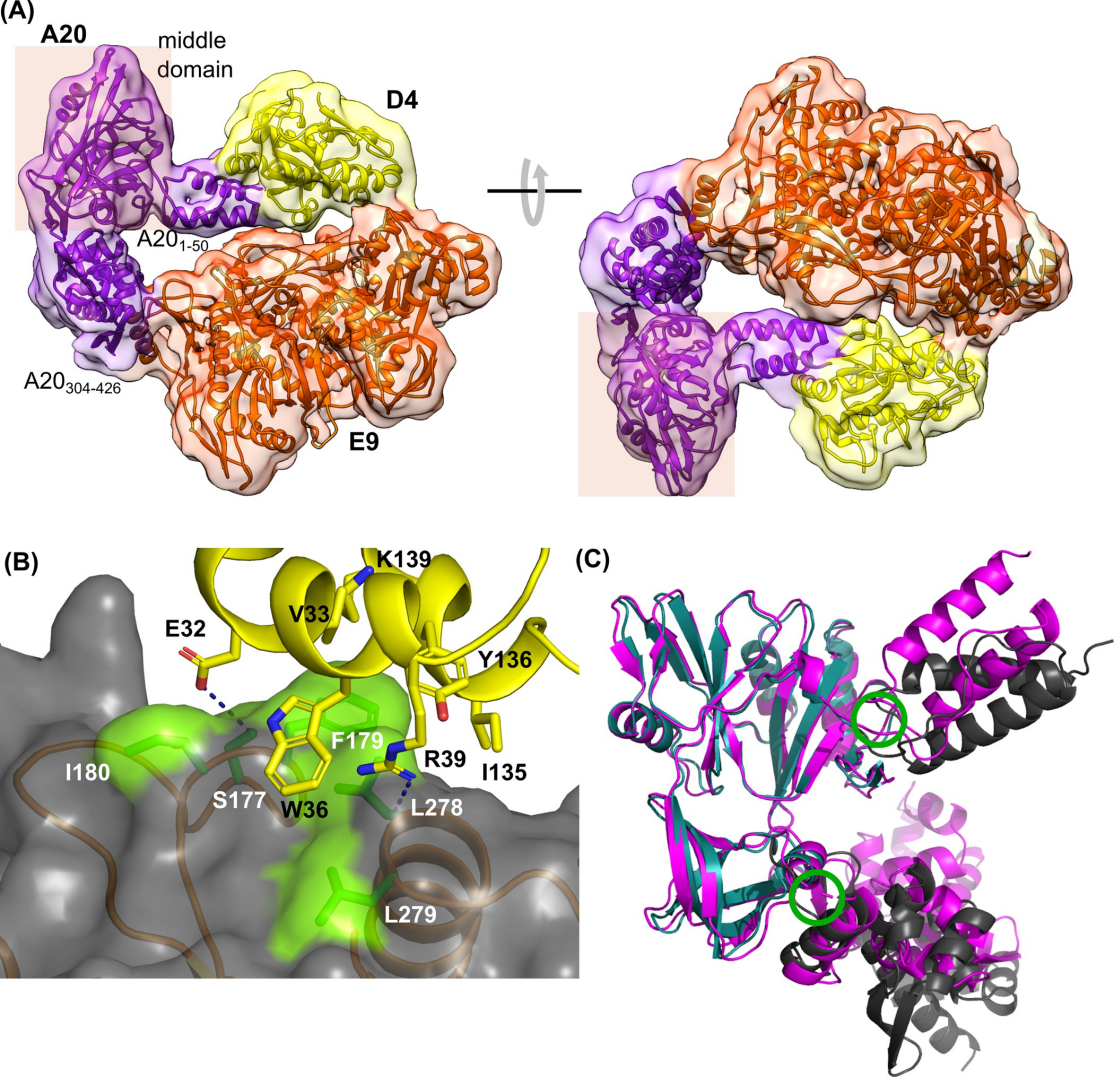
VACV E9-A20-D4 holoenzyme structure. (**A**) The refined 3.8 Å structure of the holoenzyme is displayed in cartoon representation in two views together with the electron density map before sharpening. The disordered thumb domain of E9 (res. 830–1006) is not shown. (**B**) The D4 binding site of E9 (gray) is shown in a transparent surface representation with the sidechains of the underlying contacting residues coloured in green. Contacting residues of D4 (yellow) are shown as a stick model. (**C**) Superposition of A20 in the context of the VACV polymerase holoenzyme heterotrimer (violet) with the Alphafold 2 prediction (turquoise for the middle domain, otherwise black). Green circles indicate hinges.

The E9-A20-D4 holoenzyme could be expressed with a good yield in insect cells using a single recombinant baculovirus. The double tag, a Tobacco Etch Virus (TEV) protease cleavable 6His-tag on E9 and a Strep-tag on D4, resulted in an efficient purification and led to a stoichiometric complex (Fig 1AB). SEC-MALS analysis of the purified complex yielded a single peak of the complex with a mass of 185 kDa compared to a theoretical mass of 194 kDa (Fig 1C). The efficiency of the purification step on the streptactin column allowed the simplification of the purification protocol for cryo-EM sample preparation by the omission of the TEV cleavage and second Ni-column purification step. The analysis of the E9-A20-D4 complex by cryo-electron microscopy was hampered by strong preferential orientations of the particles at the ice-air interface. One dataset showed less prominent preferential orientations, which permitted the 3D reconstruction of the complex (S1 Fig) and refinement to a resolution of 3.8 Å (Fig 2A and S1 Table). Surprisingly, the structure showed a conformation, where the processivity factor A20-D4 was folded back onto the polymerase subunit creating the D4-E9 interface described for the MPXV holoenzyme in presence of DNA (Fig 2B, [23]). The parts of the complex where previous high-resolution structures were available were very well defined so that E9 [21], the D4/A20_1-50_ complex [20] and the NMR structure of A20_304-426_ [22] could be fitted into the density. The least well-defined part of the model of A20 is the middle domain (res. 67–310) corresponding to an inactive DNA ligase composed of a catalytic subdomain followed by an Oligonucleotide/oligosaccharide-Binding (OB)-fold domain [27]. The ligase domain had been identified in the MPXV processivity factor protein A20 [23] and in the Alphafold 2 predicted protein structures of orthopoxviruses [28]. The Alphafold 2 prediction of A20 could be fitted reliably into the electron density after adjustment of the relative orientation of the domains A20_1-50_ (Fig 2C), middle domain and A20_304-426_ although for the final structure a model based on the very similar structure from MPXV has been used. The flexibility around the hinge regions creates probably a disorder of the middle domain, which limited the number of exploitable particles for 3D reconstruction and refinement (S1 Fig). The thumb domain (res. 830–1006) of E9 is not visible in the electron density showing its mobility. In the refined model of the VACV polymerase holoenzyme, the carbon-alpha positions of the middle domain of A20 superpose with 0.56 Å rms onto the corresponding MPXV structure and with 1.13 Å rms onto the Alphafold 2 model.

The E9-D4 interface mainly involves two α-helices of D4, which contribute E32, V33, W36 and R39, respectively I135, Y136 and K139, while on E9, S177, F179, I180, L278 and L279 are involved (Fig 2B). The side chains of D4 E32 and E9 S177, as well as D4 R39 and the main chain carbonyl of E9 L278 could be involved in hydrogen bonds (Fig 2B, dotted lines). This interface belongs to one of the best-defined parts of the complex with a local resolution below 4 Å (S1E Fig). The above-mentioned residues are strictly conserved according to a protein BLAST search (blast.ncbi.nlm.nih.gov) on reference sequences of orthopoxviruses.

**Fig 3:**
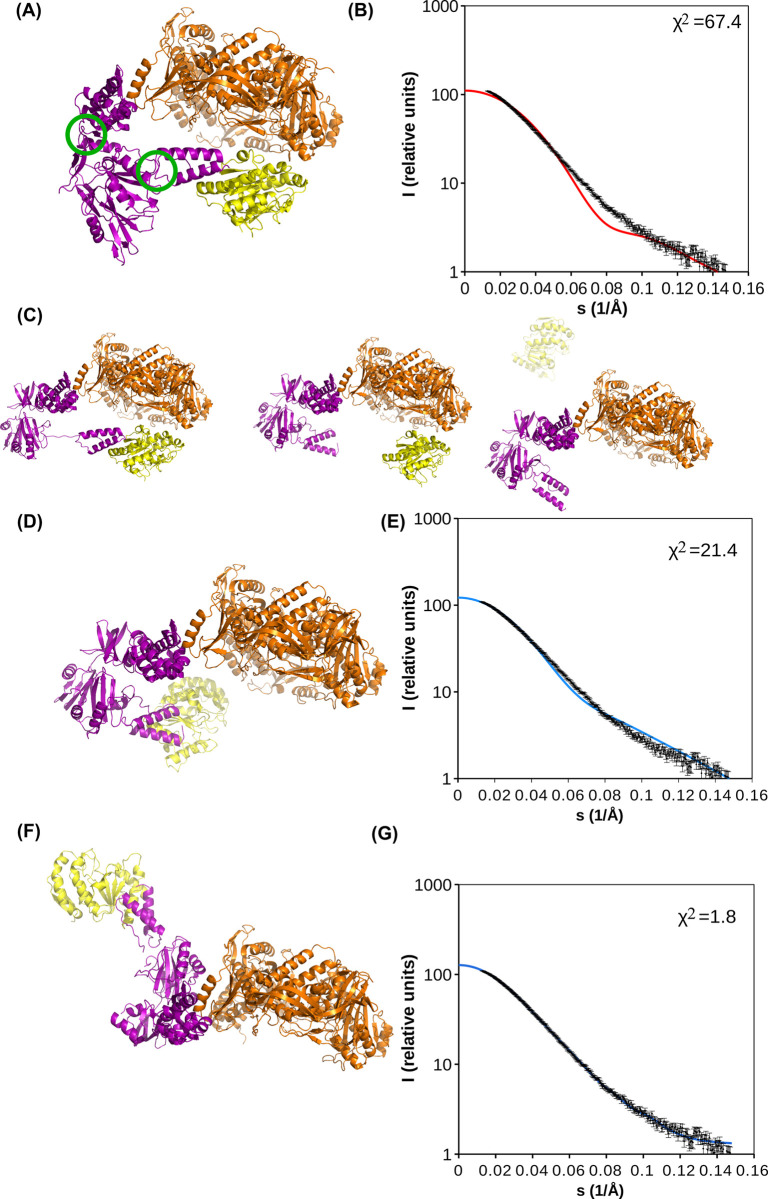
VACV E9-A20-D4 holoenzyme models and their agreement with SAXS data. Coloring scheme of the models as in Fig 2A. (**A**) Refined 3.8 Å cryo-EM structure of the holoenzyme. Green circles indicate the domain boundaries of A20, which are likely flexible hinges for the holoenzyme. (**B**) Agreement of the experimental SAXS scattering curve and the scattering curve calculated from the cryo-EM model (A) plotted with a red line. (**C**) Different types of solutions of multiple runs of Alphafold 2 on E9-A20-D4: models similar to the complex observed in cryo-EM shown in panel (A), left; models with an E9-D4 interaction similar to the one of the cryo-EM structure, but with a disrupted A20-D4 interface (middle); models with isolated D4 (right). (**D**) The middle model of (C) is used but the A20-D4 interface is restored by a repositioning of D4 according to the A20_304-426_-D4 structure [20]. (**E**) The calculated scattering curve (blue) of the model shown in (D) is compared to the experimental curve. (**F**) Model of E9-A20-D4 refined against the experimental scattering curve using Coral using the two hinges indicated in (A) and a flexible connection of the thumb domain. (**G**) The calculated scattering curve (blue line) of the refined model from Coral shown in (F) is compared to experimental scattering curve.

This compact form of the holoenzyme in absence of bound template and primer strand DNA was unexpected as SAXS data obtained from coupled size-exclusion chromatography (SEC)—SAXS experiments suggested larger dimensions of the complex (Table 1 and Fig 3A and 3B). We hypothesized initially that the compact form of the DNA holoenzyme observed in cryo-EM was an inactive form of the complex induced by the sample preparation conditions. Verification of the 2D classifications of the particles in cryo-EM gave no hint at classes with larger dimensions. In order to explore potential conformations of the complex and to assess the importance of the E9-D4 interface we first used Alphafold 2.

**Table 1:** Parameters derived from SAXS measurements.

Structural parameters of E9-A20-D4	Experimental values	Calculated from the cryo-EM model^1^
R_max_ (nm) [from P(r) function]^2^	18.0	12.6
R_g_ (nm) [from P(r) function]^3^	5.16	
R_g_ (nm) [from Guinier plot]^3^	4.97	4.18
Porod volume V_p_ (nm^3^)	350	
Molecular mass M_r_ [from V_p_] (kDa)^4^	212^5^	
Molecular mass M_r_^4^ [ML estimation from Primus] (kDa)	177–221^5^^,^^6^	

^1^ with the thumb domain modelled using the crystal structure of E9 (pdb entry 5n2e)

^2^R_max_: Maximal dimension of the molecule

^3^R_g_: Radius of gyration

^4^using a specific volume of 1.65 Å^3^Da^-1^

^5^Theoretical mass calculated from sequence: 193 857 Da

^6^p = 0.062

When Alphafold 2 was used to model the structure of the holoenzyme starting from the sequences of E9, A20 and D4, the 25 proposed models systematically predicted the E9-A20 interface whose strong interaction was described earlier [22]. However, in 19 models, the A20-D4 interface was not predicted despite the tight interaction observed experimentally [20]. Four E9-A20-D4 models were similar to the cryo-EM model with a closed conformation (Fig 3C left). The E9-D4 interaction was lost in 2 models that had an open conformation. In contrast, the E9-D4 interaction was maintained in 4 other models that presented an open conformation, but the A20-D4 interaction was disrupted (Fig 3C middle). In 15 models, D4 lost both the A20 and E9 interactions, leading to an isolated D4 subunit (Fig 3C right). In total, in 21 models A20 has a similar orientation compatible with an open conformation. Assuming that the loss of the A20-D4 interaction was unlikely fold zyme complexa functional t00E and L204K.e E9-D4 interaction, open models could be built where D4 was again associated with A20 (Fig 3D), using the known relative orientation of the two partners [20]. Such a model is already more open than the cryo-EM structure and explains the SAXS scattering curve better (Fig 3E). Using the flexibility at the two identified hinges within A20 and another hinge connecting the thumb domain to the body of E9, in a rigid-body refinement against the SAXS scattering curve, the refined model fitted almost perfectly to the SAXS data (χ^2^ = 1.82, Fig 3F and 3G), and agreed also in the maximal dimension (17.5 nm) and R_g_ (5.0 nm) with the results obtained from SAXS (Table 1). This model is certainly inaccurate, as an ensemble of structures would contribute to the scattering curve; but with the limited amount of experimental data, we discontinued further modeling and concluded that in solution more open conformations of the holoenzyme were likely to be present.

**Fig 4:**
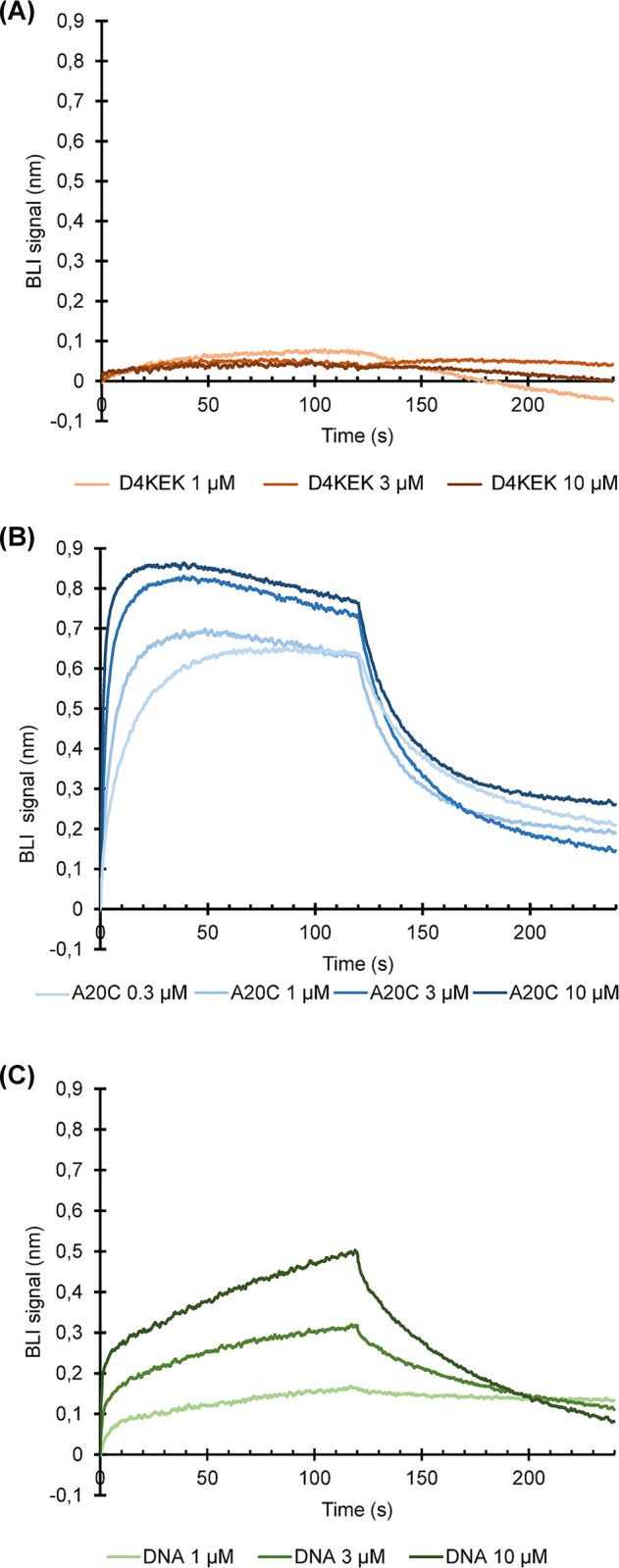
Analysis of the E9-D4 interaction by BLI. E9 is immobilized on a sensor tip using its 6His-tag. Data have been corrected for the unneglectable dissociation of 6His-tagged E9 from the sensor tip. (**A**) Interaction with a monomeric mutant of D4 (D4KEK). Controls: (**B**) Interaction with A20_304-426_ (A20C); (**C**) interaction of E9 with a dsDNA composed of a 37 base template strand with a 25 base 5’ overhang and a 12 base primer strand.

**Fig 5:**
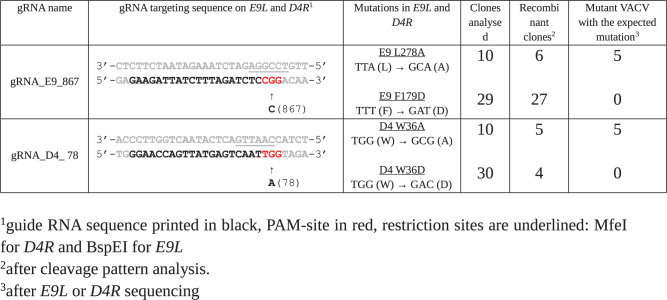
Mutant VACVs generated with the CRISPR/Cas9 system.

We next addressed the importance of the E9-D4 interface. We first tried to measure a direct interaction between the polymerase subunit E9 and a monomeric mutant of D4, D4KEK, which carries the mutations I197K, V200E and L204K. This mutant does not form dimers in solution as wild-type D4 does [29]. We chose the KEK mutant as we were concerned that the artefactual dimerization of wt D4 in absence of A20 [20] might interfere with the binding studies. The D4KEK structure, which could be determined at a resolution of 1.32 Å (S2 Table), is virtually undistinguishable from wild-type D4 (S2 Fig), except the dimerization site. As the mutations are distant from the interaction site with E9 they should not interfere with binding. Using BioLayer interferometry (BLI), it was neither possible to observe an interaction between E9 and D4KEK (Fig 4A) nor between E9 and wild-type D4 (S3 Fig). In control experiments however, the previously known interaction between E9 and A20_304-426_ [21] could be confirmed (Fig 4B) as well as the interaction of E9 with a dsDNA substrate [21] (Fig 4C). The dissociation of E9 from the Ni-NTA tip of the BLI instrument, a relatively small size of the ligand and biphasic binding kinetics precluded a full quantitative treatment of the data. We conclude that binding of D4 to E9 in the absence of DNA is too weak to be observed without A20 acting as a linker suggesting that the D4 and E9 interact only transiently. In order to address the question of whether the interaction is essential, we generated mutants of VACV using a CRISPR-Cas9 based system [30]. We targeted the most prominent residues of the E9-D4 interface, F179 and L278 of E9 and W36 of D4 (Fig 2B), with mutations to alanine or a more radical introduction of a charged aspartic acid residue (Fig 5). The approach had been validated previously on the E9-A20 interface [30], where the results were correlated with complex formation [21]. It is expected that the number of recombinant VACVs carrying the mutation decreases with the functional impairment of the mutant. The W36D mutation (Fig 5) did not allow virus recovery, whereas the mutation to alanine seemed to be better tolerated. Likewise, the E9 F179D mutation appeared to be lethal, whereas viruses carrying the L278A mutation could be recovered. The alanine mutants have a growth rate reduced by only about 20% compared to mutants carrying only a silent mutation (S4 Fig). The effect of the mutations is in agreement with the expectations from the analysis of the three-dimensional structure of the interface (Fig 2B) and we conclude that despite its weakness, the E9-D4 interaction is essential.

## Discussion

Following the mpox outbreak, several structures of the MPXV DNA polymerase holoenzyme became available, with [23,25] or without bound dsDNA substrate [24,25]. Surprisingly, the apo structure of the VACV polymerase holoenzyme (Fig 2A) believed initially to represent an inactive state induced by the cryo-EM sample preparation showed a closed conformation similar to the MPXV structure with bound template, primer strand and incoming nucleotide [23]. In contrast to the findings described by Li and co-workers [24] on MPXV holoenzyme in the absence of DNA substrates, there is no indication of the formation of a dimer of heterotrimers. A superposition of the heterotrimer of VACV onto the heterotrimer from MPXV in the absence of DNA [24] shows a near to identical overall structure (S5A Fig). Nevertheless, the middle domain of A20 differs considerably in structure and orientation, leading to a 4.6 Å rms difference for aligned Cα positions when A20 is compared to the MPXV counterpart (S5B Fig), and still a 2.3 Å rms difference when only the middle domains are superposed. The electron density of the middle domain of MPXV A20 from Li and coworkers seems far below the overall resolution of 3.1 Å. Therefore, it is not easily interpretable, possibly explaining why 56 residues (res. 46–101) from the linker between the N-terminal domain and the ligase subdomain have not been modelled. We assume that our structure of the VACV middle domain is more analogous to the real structure, as it closely resembles the MPXV A20 middle domain presented by Peng and coworkers, and the Alphafold 2 model (Fig 2C). Additionally, our structure fits the electron density deposited in EMDB entry EMD-34887 as well as the model of Li and co-workers [24] (pdb entry 8hm0).

The residues of the E9-D4 interface are located in well-defined parts of the structures, and are the same for the apo form of the MPXV and VACV holoenzymes. Differences in sidechain conformations are within the error margin resulting from the limited resolution of the two structures.

**Fig 6:**
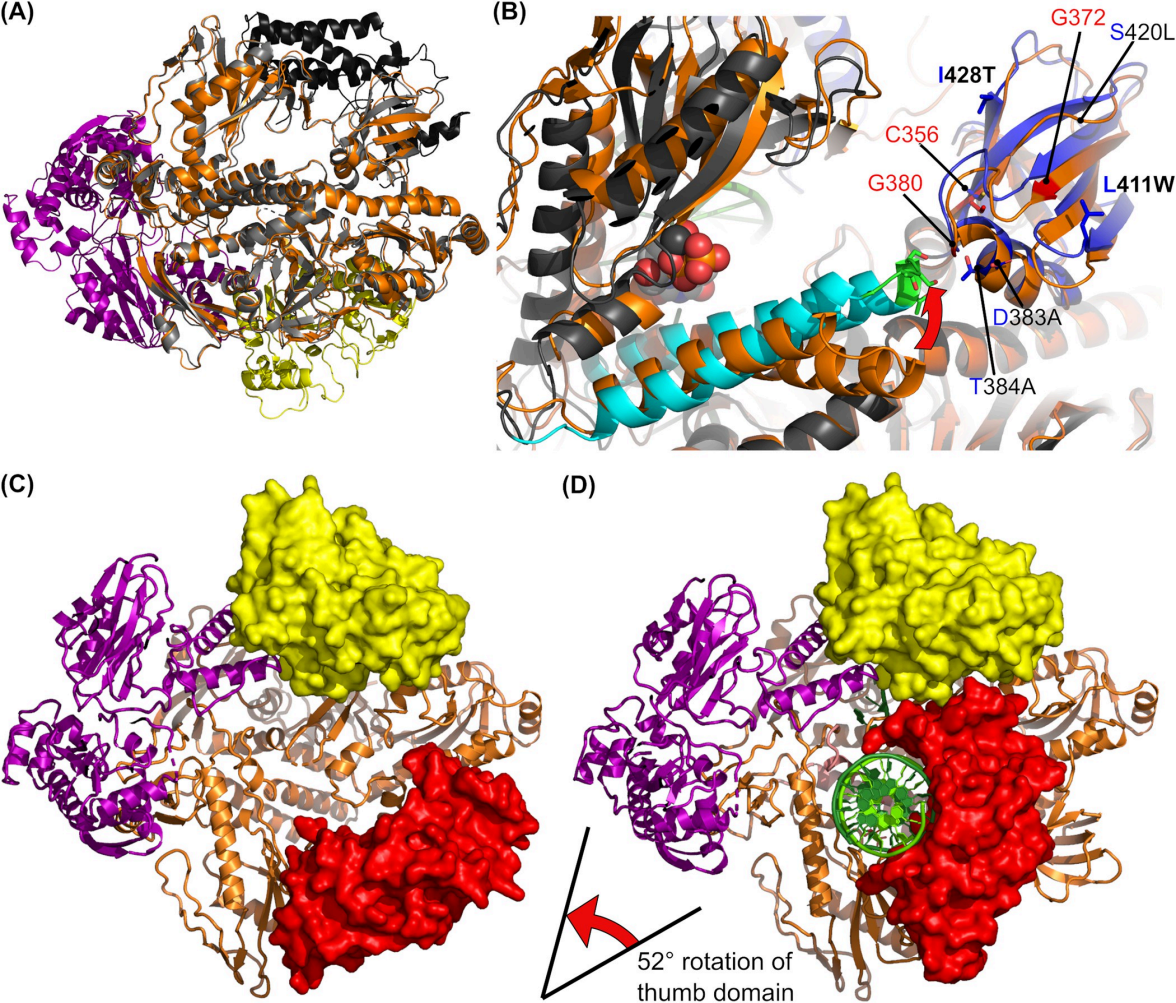
Movements within the holoenzyme complex. (**A**) VACV E9 crystal structure (pdb entry 5n2e [21], grey, thumb domain in black) superposed onto the refined structure of the VACV E9-A20-D4 complex (colours as in Fig 2A). (**B**) Close-up of the fingertip and the insert 2 region of the apo form of VACV holoenzyme (orange) superposed onto the DNA-bound MPXV E9 (gray, finger domain in cyan, residues of the fingertip in stick representation and highlighted in green, insert 2 domain in blue) with an incoming dCTP nucleotide (spheres) [23]. A red arrow shows the direction of the movement of the fingertip upon nucleotide binding. Residues involved in PAA resistance of VACV polymerase [31] are labeled and shown in red. Residues of insert 2 differing between MPXV (GenBank accession ON755039, [2]) and VACV are shown in stick representation and labelled. Residue labels of VACV E9 are printed in black, the corresponding residues from MPXV are printed in blue and additionally in bold if the mutations have been acquired recently. (**C**) VACV E9-A20-D4 cryo-EM structure where the invisible thumb domain is added according to the crystal structure [21]. For clarity, D4 (yellow) and thumb domain (red) are shown in surface representation. (**D**) The same view of the MPXV holoenzyme with bound DNA (pdb entry 8hg1 [23]). Domains are colored as in (C); the DNA is shown in green.

A comparison of the cryo-EM structure of the E9-A20-D4 complex with several crystal [20,21] and NMR structures [22] of individual domains and proteins showed the absence of any significant structural rearrangements upon complex formation. The thumb domain of E9 is disordered in the cryo-EM structure, whereas it is stabilized in the crystal structure due to a contact with a neighboring molecule. This also explains why its orientation is very different from those commonly observed ones within family B polymerases [21]. For the body of E9, the Cα backbone in the context of the holoenzyme moves by less than 1 Å rms compared to the structure of isolated E9 (Fig 6A).

Our three-dimensional structure of the VACV holoenzyme enabled us to revisit previous work that had identified several temperature-sensitive (ts) mutants of A20, which compromise processive DNA replication, while conserving the distributive action of E9 (S3 Table). Punjabi and coworkers [32] analyzed previously identified ts mutants and created a number of clustered charged-to-alanine mutants. Similar charged-to-alanine VACV mutants have been analyzed for viral growth by Ishii and co-workers [33] (S3 Table). The mutant Dts48 is carrying a single G84E mutation [34] situated in the connection between N-terminal and middle domain that might affect the flexibility of A20 or could potentially lead to misfolding of A20 at the non-permissive temperature and interfere with the assembly of a functional holoenzyme complex. The charged-to-alanine mutants A20-ER (185ER to AA) [32] with mutations located in the ligase subdomain and A20-4 (177DDE to AAA) [32] were inactive in processive DNA synthesis. Again, this is probably explained by misfolding as D178 of the 177DDE to AAA mutant caps a small helix and E185 of the 185ER to AA mutant is involved in structural hydrogen bonds. Why the more radical A20-ER-5 mutant only led to a ts phenotype remains obscure, but nonetheless agrees with the results of Ishii et *al*. [33]. Globally, there is a compelling correlation between the effect of the mutation in processive DNA synthesis and viral growth and the structural role of the residues (S3 Table). However, it is important to note that the introduction of multiple alanine residues at the same time may affect non-specifically the secondary structure leading to inactive or ts phenotypes.

The dimer of trimers observed by Li and coworkers [24] in pdb entry 8hlz is intriguing. The assembly of this MPXV hexamer is formed by two extended E9-A20-D4 complexes interacting around a quasi-2-fold axis, using in *trans* the E9-D4 interface observed in the heterotrimer (S5C Fig, top). This leads to additional contacts between the thumb domain of E9 and A20. In order to explain the transition from a dimer of trimers to a compact trimer with an E9-D4 contact in *cis*, the author proposed a dissociation of A20 from D4 in the dimer of trimers and rebinding of A20 to the other D4 molecule leading to compact trimers. This model can be refuted as we showed that the E9-D4 interaction is transient with less buried surface (380 Å^2^ analyzed with PDBePISA [35] in VACV, 340 Å^2^ on average for MPXV, pdb entry 8hlz) than the A20-D4 interaction (830 Å^2^), or, for comparison, the E9-A20 interface (670 Å^2^). In addition, the A20-D4 interface appears to be extremely important, as it is not possible to produce A20 on its own [20]. Instead, we propose a transition of the hexamer to the trimer involving a dissociation of D4 from E9 in the hexamer, a change of the conformation of A20 and rebinding to E9 in *cis* leading to two compact trimeric complexes (pdb entry 8hm0, S5C Fig, bottom).

The DNA-free cryo-EM structure of VACV E9-A20-D4 (Fig 3A) is smaller than suggested by the SAXS curve from the holoenzyme complex in solution (Fig 3B), which indicates a greater radius of gyration (4.97 nm vs. 4.18 nm) and a larger maximal dimension (18.0 nm vs. 12.6 nm, as outlined in Table 1). SAXS results differed from previous data [18], from which an even more elongated structure of the complex (R_g_ = 7.12 nm) was calculated. Reprocessing of the previously published data showed the contribution of aggregates to the scattering interfering with a reliable determination of the radius of gyration. In fact, at that time the SAXS measurements were not coupled to SEC, in contrast to the current data.

For A20, Alphafold 2 predicted 3 independent domains and identified two hinge regions (Fig 2C) suggesting flexibility of the protein. The first hinge is located within the linker (res. 50 to 67) connecting the N-terminal domain to the middle domain. The second hinge is located around residue 310, in the connecting region between the middle domain and the C-terminal domain A20_304-426_. This flexibility made it possible to fit the Alphafold 2 model of A20 into the cryo-EM electron density without other modifications (Fig 2A and 2C). A model, which was refined against the SAXS data after the introduction of flexible linkers, was found to be in good agreement with the experimental scattering curve (Fig 3F and 3G). Although in reality, a range of different conformations, including closed ones, is expected.

Another argument in favour of the presence of open and closed conformations in solution, is that the SAXS scattering curve can be explained by a combination of an extended VACV holoenzyme model based on the MPXV hexamer structure [24] and our compact holoenzyme structure (S6 Fig). The maximal dimension of 18 nm of the extended model (S6A Fig) corresponds to the maximal dimension of the VACV holoenzyme in solution determined by SAXS (Table 1). The mixture of compact (S6A Fig, 42%) and the extended (S6B Fig, 58%) VACV holoenzyme fitted the observed SAXS scattering curve (S6C Fig) almost as well as the Coral-fitted model (Fig 3F and 3G).

The conformational variability of the holoenzyme has been explored further by Alphafold 2 predictions of the complex structure. The predictions were either similar to the closed form (Fig 3C, left) or yielded open forms (Fig 3C, middle and right).

Based on these three lines of evidence we concluded that in the absence of an interaction with DNA, open conformations of the VACV DNA polymerase holoenzyme are present in solution. We further studied the importance and the strength of the E9-D4 interface. Previous publications had identified both the E9-A20 interaction and the A20-D4 interaction, but never an E9-D4 interaction [14]. Indeed, the occluded surface area (380 Å^2^) of the E9-D4 interface is much smaller than those observed for A20-D4 (830 Å^2^) and E9-A20 (670 Å^2^). In contrast to the established interaction of E9 with A20_304-426_ and with DNA (Fig 4B and 4C) it was not possible to measure an affinity of the direct interaction of E9 and D4 (Fig 4A). This suggests that in solution, the interaction observed in the cryo-EM structure (Fig 1A and 1B) is weak. Still, site-directed mutagenesis results (Fig 5) showed that viruses radically mutated in key residues of the E9-D4 interface are not viable, whereas less drastic mutations seem to be tolerated. This highlights the importance of the E9-D4 interaction. These results agree with biochemical work by Peng and coworkers [23] who analyzed the effect of mutations in the interface on processive DNA synthesis. D4 W36 was found to play a central role, as the W36A mutation led to a much reduced activity. Furthermore, DNA synthesis activity was completely lost, when the W36A mutation was combined with the R39A mutation whereas the D4 mutation of N165A in vicinity of E9 did not have any effect.

Our structure of the apo form of the holoenzyme shows a conformation, which is similar to the DNA-bound MPXV holoenzyme structure of Peng and co-workers [23] whose structure allowed to understand the role of the E9-D4 interaction (Fig 6D). This interaction leads to the formation of a ring entrapping the template strand. In the DNA-bound MPXV holoenzyme structure the neo-synthesized primer strand induces a movement of the thumb domain, which closes upon the dsDNA leading to a rotation of 52° relative to the orientation of the thumb in the VACV E9 crystal structure (Fig 6C, [21]). Similar but less pronounced rotations, on the order of 15°, between free and DNA template and primer strand bound structures, are observed for other family B polymerases, such as the herpes simplex virus polymerase [36,37]. This movement positions a large insertion in E9 (residues 896–925) close to D4, assigning indirectly a role for these residues, which are inserted into in the 4-helix bundle of the thumb domain and which are conserved within the E9 polymerase of poxviruses. The insertion appears to form a platform indirectly supporting D4. The direct E9-D4 interactions are restricted to the previously analyzed hydrophobic interface around W36 of D4 (Fig 2B). The thumb domain closes onto the neo-synthesized dsDNA, whereas the E9-D4 contact encircles the template DNA assuring the processivity of the polymerase.

Lastly, a structural difference between apo and DNA bound holoenzyme is the movement of the fingertip observed in the MPXV structure in presence of DNA and an incoming dTTP nucleotide, which is characteristic for class B polymerases [38,39]. In the quest for a role of the insert 2 domain (res. 356–432) we proposed a contact between insert 2 and the fingertip [21]. Indeed, it contacts insert 2 in the MPXV structure (Fig 6B). The role of this interaction is not understood but insert 2 carries resistance mutations (shown in red in Fig 6B) against phosphonoacetic acid (PAA), a pyrophosphate analog inhibiting VACV polymerase. Insert 2 also clusters sequence differences between VACV Copenhagen and MPXV (Zaire-96-I-16, NCBI NP_536484.1, Fig 6B, blue labels), including some recently acquired mutations in the IRBA22-11 strain of MPXV [2] (Fig 6B, bold labels).

The model presented by Peng and co-workers [23] provided information regarding the position of D4 on the template strand although there is a lack of density corresponding to the DNA backbone between E9 and the D4 active site. The same observation is made by Wang and collaborators ([13], pdb entry 8wpe). However, they also observe the continuation of the template strand in one of their structures (pdb entry 8wpf) using a dU base at the -7 position. Here, an uninterrupted DNA backbone with an abasic site present at the level of the UNG active site has been observed. This confirms the uracil excision at the level of the template strand and indeed, stalling of the polymerase upon the detection of a uracil residue in the template had been observed [34].

Numerous questions are remaining open: Up to date, there is no information on the repair mechanism taking over after the generation of the abasic site by D4 and the stalling of the replication fork [34]. In addition, the structural basis for a possible uracil excision in the neo-synthesized strand [34] has still to be elucidated and may be due to another holoenzyme complex acting in *trans*. Future structural and biochemical work is finally needed in order to cast light on the role of E9 as recombinase. In this role, E9 appears to use only the exonuclease site and neither the polymerase active site nor the A20-D4 processivity factor [17]. Additionally, this could still involve unknown conformations of the holoenzyme.

## Materials and methods

### Construction of the baculovirus expressing E9-A20-D4

The MultiBac baculovirus/insect cell expression system [40] was used in order to express the VACV DNA polymerase holoenzyme using sequences of *E9L*, *A20R*, and *D4R* of VACV strain Copenhagen (GenBank accession number M35027.1). Gene synthesis and cloning was performed by GenScript. *E9L* was inserted into the pUCDM vector. Recombinant E9 contains a 6His-tag and the TEV protease cleavage site sequence fused to its N-terminus. *A20R* and *D4R* (D4 carrying an N-terminal Strep-tag) were both cloned into the pFL plasmid. In consequence, E9 carries an N-terminal TEV-cleavable 6His-tag with the sequence MSYYHHHHHH DYDIPTTENL YFQ↓GAMDP, which replaces the N-terminal methionine. D4 carries an N-terminal Strep-tag with the sequence MASWSHPQFE KSGGGGGLVP RGSA before the N-terminal methionine residue.

*E9L*, *A20R*, and *D4R* were then combined by *in vitro* fusion of acceptor (pFL) and donor (pUCDM) plasmid derivatives using Cre recombinase [41]. The resulting construct was used to transform DH10EMBacY bacteria provided by the Eukaryotic Expression Facility (EEF, EMBL Grenoble). Bacmid carrying the VACV genes was extracted, purified and transfected into *S*. *frugiperda* (*Sf21*) cells. Transfection of 10^6^ cells in a 6-well plate was performed using 5 μl of transfection agent (X-tremeGENE HP Reagent, Roche), 10 μL of bacmid and 85 μL of SF900II-SFM medium (Gibco). *Sf21* cells were incubated for 48 h at 27°C. The virus-containing supernatant (V_0_) was recovered. The viral stock for protein expression was prepared as follows: 25 mL of *Sf21* cells at 0.5×10^6^ cells/mL were infected with 3 mL of V_0_. Cells were maintained at 0.5·10^6^ cells/mL until cell growth stopped. After centrifugation at 1000×g for 3 min, the supernatant containing recombinant baculovirus was recovered. Viral stocks were stored at 4°C, protected from light.

### Protein expression and production

E9-A20-D4 has been purified using a long protocol or a simplified protocol. Frozen cell pellets from 2 L of infected High Five (Thermo Scientific) insect cell culture were resuspended in 50 mM Tris-HCl pH 8.5, 300 mM NaCl, 10% glycerol, 5 mM imidazole, 5 mM β-mercaptoethanol (buffer A) with Complete protease inhibitor cocktail (Roche) and 2 mg DNAse I from bovine pancreas (Sigma-Aldrich) and lysed in a Potter homogenizer (B. Braun, Melsungen, Germany) using 20 strokes or by sonication (5 min, Cycle: 0.5, Amplitude: 70%; Labsonic, Satorius). After centrifugation (58 000×g at 4°C) for 20 min, the supernatant was loaded on a 5 mL His-trap FF Crude column (Cytiva) using a peristaltic pump. The column was washed with 20 mL of buffer A and eluted with 20 mL of the same buffer containing 180 mM imidazole.

The sample was concentrated on a Vivaspin 30 kDa centrifugal concentrator (Sartorius) to 3 mL for buffer exchange with an Econo10 DG column (Biorad) equilibrated in buffer A and eluted with 4 mL of the same buffer. 1/100 (w/w) TEV protease were added and incubated overnight at room temperature.

The digested complex was diluted to 15 mL with buffer A and loaded on same Ni-column equilibrated in buffer A. The column was washed with 15 mL of buffer A first, followed by 20 mL of 20 mM imidazole in buffer A. Complex-containing fractions were pooled, concentrated to 3.5 mL and diluted with 50 mM Tris-Hcl pH 8.5 for a final NaCl concentration of 200 mM. A 1.5 mL Streptactin (IBA Lifesciences, Göttingen, Germany) column was equilibrated in 100 mM Tris-HCl pH 8.5, 200 mM NaCl (buffer C), the sample was loaded and the column washed with 5 mL buffer C. The complex was eluted in five 1 mL fractions using buffer C with added 5 mM desthiobiotin (IBA Lifesciences). After addition of TCEP to 1 mM, complex containing fractions were concentrated to 0.8 mL and injected on a Superdex S200 10/300 (Cytiva) column equilibrated in 20 mM Tris-HCl pH 8.5, 200 mM NaCl and 1 mM TCEP. The peak fractions were concentrated to ≈ 2 mg·mL^-1^ for further use in structural studies.

A simplified protocol has been used for the cryo-EM studies. It skips the TEV digestion and the second Ni-column and used the buffer-exchanged protein directly on the streptactin column omitting also the size exclusion chromatography step.

### Multiple Angle Light Scattering (MALS)

SEC was performed with a Superdex 200 10/300 GL (GE Healthcare) equilibrated in 50 mM Tris-HCl pH 7.5, 100 mM NaCl. The run was performed at 20°C with a flow rate of 0.5 mL·min^−1^. 50 μL of a protein solution at a concentration of 2 mg·mL^−1^ were injected. On-line MALS detection was performed with a DAWN-EOS detector (Wyatt Technology Corp., Santa Barbara, CA) using a laser emitting at 690 nm. The protein concentration was measured inline by refractive index measurements using a RI2000 detector (Schambeck SFD) using a refractive index increment dn/dc = 0.185 mL·g^-1^. Data were analyzed and weight-averaged molecular masses (Mw) were calculated using the software ASTRA V (Wyatt Technology Corp., Santa Barbara, CA) as described [42].

### SEC-SAXS

SAXS measurements were done on BM29 of ESRF at a ring current of 200 mA. 45 μL of E9-A20-D4 complex with a cleaved His-tag at 2 mg·mL^-1^ in 25 mM Tris-HCl pH 7.5, 300 mM NaCl were injected onto a Superdex S200 3.2/100 (Cytiva) size exclusion column. Runs were performed at a flow rate of 0.1 mL·min^-1^ and 2000 frames of 1 s were collected using a Pilatus 1M detector (Dectris). Individual frames were processed automatically and independently within the EDNA framework yielding radially averaged curves of normalized intensity versus scattering angle s = 4πsinθ/λ [43]. Frames corresponding to the elution of E9-A20-D4 were identified in iSPyB [44], merged and analyzed further using Primus of the ATSAS package [45].

### SAXS data treatment

SAXS data were analyzed with different programs of the ATSAS package [45]. Scattering curves of models were calculated and compared to the experimental scattering curve using Crysol. Rigid-body modeling with Coral used 4 bodies (D4+A20_1-54_, A20 middle domain (res. 56–310), A20_312-426_+E9_1-830_, E9 thumb domain E9_832-1006_) based on the E9-A20-D4 model from cryo-EM. A linker residue has been introduced at the position of the hinges of A20 and the connection of the thumb to the body of the polymerase. The relative contributions of extended and compact forms of the holoenzyme were obtained with Oligomer [46].

### Alphafold 2 predictions

Predictions of the complex structure used an Alphafold 2 [47] installation on the CCRT-HPC TOPAZE super calculator from the CEA (https://www-ccrt.cea.fr). The sequences of the three proteins of E9-A20-D4 have been provided and 5 runs of 5 models have been generated resulting in an ensemble of 25 predicted conformations of the trimeric complex.

### Cryo-EM of the apo form of the E9-A20-D4 holoenzyme

The purification omitted TEV cleavage and the 2^nd^ Ni-column purification step and E9-A20-D4 complex was used directly after the Streptactin column purification step and concentrated in a Vivaspin 6 concentrator with 30 kDa cut-off to 1.7 mg·mL^-1^. The sample was centrifuged for 20 min at 13 000 rpm in an Eppendorf centrifuge. 5 μL of a buffer (80 mM Tris-HCl pH 7.4, 20 mM EDTA) were added to 15 μL of complex. In the Vitrobot chamber with 100% humidity at 4°C, 4 μL of sample were applied for 30 s to 1.2/1.3 holey carbon on 300 mesh copper grids (Quantifoil), followed by 5 s blotting with blot force 20 and plunge freezing in liquid ethane with a Vitrobot IV (FEI, Thermo Scientific). Grids were glow discharged for 90 s in air at 13 Pa in a Plasma Cleaner model PDC-002 (Harrick Plasma, Ithaca, NY, USA).

### Cryo-EM data collection and processing

Data were collected with EPU on the FEI Krios electron microscope (Thermo Scientific) of the Rudolf-Virchow-Zentrum, Würzburg, Germany. Statistics are given in Table 2. Data were processed on the EMBL-IBS computing cluster with a SBGrid [48] software installation. Motion correction used Relion 4.0 [49]. The downstream data processing used Cryosparc [50] using the pipeline shown in S1 Fig. The final step of non-uniform refinement refined also tilt and per group CTF parameters. Maps and models were displayed with Chimera [51], which was also used for the docking of the existing structures into the cryo-EM map: (A20_1-50_-D4 complex, pdb entry 4od8, E9-A20_304-426,_ PDB-DEV database accession code: PDBDEV_00000075, MPXV middle domain from pdb entry 8hg1 [23]). Coot [52] was used to model the sequence of VACV A20 onto the MPXV A22 structure and for the adjustment of the linkers between individual domains. The structure was refined using Phenix real-space refinement [53] at a resolution of 3.8 Å against a map sharpened with a temperature factor of -160 Å^2^. Statistics of the final model are given in S1 Table.

**Table 2:** Cryo-EM data collection statistics.

Parameter	Cryo-EM data collection E9-A20-D4
Microscope	Titan Krios G3 (Thermo Fisher)
High tension (kV)	300
C_s_ (mm)	2,7
Detector	Falcon III camera
Mode	Counting
Energy filter	No
Magnification	42000 x
Data collection date	28/2/2022
Calibrated pixel size	1.064 Å
No. of frames per movie	47
Nominal defocus range (μm)	- (1.4–2.4)
Dose par frame (e·Å^-2^)	1.45
Dose Rate (e·px^-1^·s^-1^)	1.03
Drift rate (nm·s^-1^)	< 0.04
No. of movies	1376

### Biolayer interferometry (BLI) measurements

BLI measurements were performed on a BLItz instrument (Forté Bio) with NTA biosensors (Sartorius) using the following protocol after at least 20 min initial rehydration: baseline measurement 60 s, E9 loading 180 s, D4 or A20 or DNA association 180 s, dissociation 180 s, regeneration 30 s, baseline 60 s, nickel loading 60 s, baseline 30 s. Running buffer was 20 mM HEPES pH 7.5, 100 mM NaCl, 0.05% TWEEN 20. E9 with an uncleaved His-tag [21] was used at 0.1 mg·mL^-1^ for loading; analyte concentrations varied from 0.03 to 10 μM, regeneration used 10 mM glycine pH 1.7 buffer and nickel loading used 10 mM NiCl_2_. Data were baseline corrected, normalized for the quantity of E9 bound to the chip and corrected for dissociation of His-bound E9 using Excel (microsoft.com).

### D4-KEK mutant

In order to obtain a D4 construct, which does not dimerize at high concentration as described by Schormann and co-workers [29], we modified residues in the D4-D4 interface. Hydrophobic residues I197, V200 and L204 involved in the D4-D4 and D4-A20 interfaces were changed to their charged counterparts in the human UNG protein, which was known to be strictly monomeric. The “humanized” mutant D4 I197K-V200E-L204K (D4KEK) was obtained using the previously described pPROEX-D4 plasmid [20] mutagenized by PCR amplification of the full plasmid using primers ttCGTTGATt tTTTCAAATG ATCTATCTTT CTCG and TTACTGGAAa aAGACAACAA GGTACCTATA AATTGG (lower letters indicate the introduced mutations) followed by recircularization by ligation. The resulting construct contains an N-terminal 6His-tag and a TEV cleavage site. D4KEK was expressed and purified using the same protocol as described for wild-type D4 [20]. Initial crystallization conditions of D4KEK were obtained using the EMBL HTX platform and were refined manually to 22% PEG4000, 100 mM LiSO_4_, 100 mM Hepes pH 7.5. Diffraction data were collected on the SOLEIL PX1 beamline on 25/07/2019. Data were reduced with XDS [54], analyzed with AIMLESS [55] and molecular replacement solution was found with MOLREP [56] using the wild type D4 structure (chain B of pdb 4od8). The structure was refined to 1.32 Å resolution using cycles of refinement with REFMAC [57] and manual building with COOT [52]. The final model superposes very well on wt D4 (0.25 Å rmsd on Cα atoms) and statistics are given in S2 Table.

### Production of recombinant VACV carrying mutations in E9L and D4R

The production of mutant VACV using the CRISPR/Cas9 mediated homologous recombination was described in Boutin *et al*. [30]. Briefly, 5×10^6^ CV-1 cells were infected with VACV at a MOI of 0.02 in DMEM supplemented with 0.5% *v/v* FCS for 1 h at 37°C in 5% CO_2_ atmosphere. Cells were then electroporated using the Neon Transfection System (Thermofisher) as follows: cells were washed once in 1× phosphate-buffered saline (PBS), harvested by trypsinization, and resuspended in 100 μL of the kits electroporation buffer R (1×10^7^ cells/mL). Cells (100 μL) were mixed with 2.25 μg of each plasmid: pCMV-Cas9ΔNLS, a donor vector carrying the mutated *E9L* or *D4R* and pU6-gRNA (Sigma) encoding gRNA targeting *E9L* or *D4R* (Fig 5). The cell/DNA mixture was aspirated into a 100 μL Neon tip and submitted to two electric pulses at 1050 V for 30 ms. Cells were then seeded in six-well plates containing warm DMEM supplemented with 10% FCS and incubated for three days at 37°C in 5% CO_2_. After a single freeze-thaw cycle, the viral suspension was recovered and diluted in DMEM before infection of Vero cells seeded in six-well plates. After 1 h of adsorption, the residual inoculum was removed and replaced with DMEM supplemented with 10% FCS and 0.6% agarose. The plates were incubated at 37°C in 5% CO_2_. Three days post-infection, 10 to 20 individual plaques were picked and used to infect Vero cells seeded in 96-well plates for two days. For each clone, the viral DNA genome was extracted using the QIAamp DNA mini kit (Qiagen). Specific genomic regions of *E9L* or *D4R* were PCR-amplified using the following primers: E9-F: 5’CTAACAAAGA GCGACGTACA AC3’, E9-R: 5’GAAGCCGTCG ATAGAGGATG3’ and D4-F: 5’CTATAGGACC TTCCAACTG3’, D4-R: 5’CCTTGAGCCC AATTTATAGG3’, respectively. PCR amplicons were purified using a QIAquick purification PCR kit (Qiagen) and digested with BspEI (*E9L*) or MfeI (*D4R*) restriction enzymes (NEB). Digested products were separated by 0.8% agarose gel electrophoresis and visualized with ethidium bromide. Mutant viruses were further characterized by Sanger sequencing of the *E9L* or *D4R* gene to ensure the presence of the expected mutations.

### Growth kinetics of mutant VACV in vitro

Twenty-four well plates containing 8×10^5^ Vero cells were infected with VACV at a MOI of 0.05 in DMEM at 37°C in a 5% CO_2_ atmosphere. At 1 h post infection (hpi), the viral inoculum is removed and fresh media containing 2.5% v/v FCS was added and the plates incubated at 37°C. Cells were harvested at 8, 24, 48, and 72 hpi. Virus yield was determined by titration of the virus on Vero cells as described [58].

## Supporting information

S1 Table Cryo-EM map and model statistics. (PDF)

S2 Table Data collection and model statistics for the D5KEK crystal structure. (PDF)

S3 Table Structure-activity relationship of previously described charged-to-alanine mutants in A20. (PDF)

S1 Fig Cryo-EM structure determination. (PDF)

S2 Fig The KEK mutant of VACV D4. (PDF)

S3 Fig Analysis of the E9–wt D4 interaction by BLI. (PDF)

S4 Fig Growth kinetics of VACV mutated in the E9-D4 interface. (PDF)

S5 Fig Comparison of the structure of the apo form of the VACV polymerase holoenzyme with the ones of MPXV. (PDF)

S6 Fig Explanation of the SAXS curve of the VACV holoenzyme using 2 conformations. (PDF)
