# Supplementary material for: Structure and flexibility of the DNA polymerase holoenzyme of vaccinia virus
Source: PLoS Pathog. 2024 May 20;20(5):e1011652. doi: 10.1371/journal.ppat.1011652 (PMC11142717; doi:10.1371/journal.ppat.1011652)
Supplement: S1 Table — (PDF) [file ppat.1011652.s001.pdf]

S1 Table. Cryo-EM map and model statistics

**Structural parameters**	E9-A20-D4 holoenzyme without thumb domain
Resolution for FSC = 0.143 (Å)	3.8
Map Wilson factor (Å^2)	180
Number of non-H atoms	11933
MolProbity	
Model map correlation (masked)	0.54
MolProbity score	2.9
Clash score	19.8
Rms deviation from ideality, bonds (Å)	0.004
Rms deviation from ideality, angles (°)	0.59
Ramachandran plot outliers (%)	0.3
Ramachandran plot allowed (%)	6.3
Ramachandran plot favored (%)	93.5
