# Supplementary material for: Structure and flexibility of the DNA polymerase holoenzyme of vaccinia virus
Source: PLoS Pathog. 2024 May 20;20(5):e1011652. doi: 10.1371/journal.ppat.1011652 (PMC11142717; doi:10.1371/journal.ppat.1011652)
Supplement: S2 Table — (PDF) [file ppat.1011652.s002.pdf]

S2 Table. Data collection and model statistics for the D4KEK crystal structure

	D4KEK overall	D4KEK outer shell
**Data collection :**		
Instrument	SOLEIL PX1	
Wavelength (Å)	0.97856	
Space group	I4	
Cell dimensions<br>a, b, c (Å)<br>α, β, γ (°)	 161.67 161.67 39.28<br>90 90 90	
Resolution range	40.42-1.32	1.34-1.32
R_{merge}	0.05	1.134
I/σI	23.4	1.9
Completeness (%)	99.5	90.2
Total reflection	1589908	63507
Unique reflection	119652	5311
CC(1/2)	0.999	0.844
**Refinement**		
R_{work}/R_{free}	0.129 / 0.165	
No non-H atoms	4651	
No water	738	
RMSD bond length (Å)	0.017	
RMSD bond angle (°)	2.118	
Ramachandran plot outlier (%)	0.55	
Ramachandran plot allowed (%)	2.20	
Ramachandran plot favored (%)	97.25	
