# Supplementary material for: Structure and flexibility of the DNA polymerase holoenzyme of vaccinia virus
Source: PLoS Pathog. 2024 May 20;20(5):e1011652. doi: 10.1371/journal.ppat.1011652 (PMC11142717; doi:10.1371/journal.ppat.1011652)
Supplement: S3 Table — (PDF) [file ppat.1011652.s003.pdf]

**S3 Table. Structure-activity relationship of previously described charged-to-alanine mutants in A20**

| | **Ishii *et al.*, 2001** | | | |
|---|---|---|---|---|
| **Name** | **First residue** | **Targeted res.** | **Activity/growth** | **Explanation** |
| | 62 | DEVKNK* | no effect | Linker between N-terminal and middle domains |
| | 108 | DDMR | no effect | Surface of ligase subdomain |
| | 167 | EIEIEED | inactive | Not obvious, affecting probably secondary structure |
| | 177 | DDE | weak ts | Helix capping by D178 |
| | 185 | ERSFDDK | stringent ts | Structural hydrogen bonds of E185 |
| | 204 | ELRR | inactive | Structural role of R207 |
| | 224 | KVDR | inactive | Not obvious, affecting probably secondary structure |
| | 248 | KDVDH | stringent ts | Structural role of H353 |
| | 255 | RSKVREH | inactive | Not obvious, K257 and E260 may have structural role, could affect binding to H5 (Wang *et al.*, 2023) |
| | 265 | KVKKK | weak ts | K269 has structural role in the OB domain - ligase subdomain interface |
| | 345 | KRKIK | weak ts | Surface of C-terminal domain, affecting potentially secondary structure |
| | | | | |
| | **Punjabi *et al.*, 2001** | | | |
| Dts48 | 84 | G84E | stringent ts | Connection between N-terminal and middle domain affecting potentially flexibility |
| 1 | 62 | DEVK | no effect | Linker between N-terminal and middle domains |
| 2 | 108 | DDMR | no effect | Surface of ligase subdomain |
| 3 | 171 | EED | no effect | Surface residues in loop |
| 4 | 177 | DDE | inactive | Helix capping by D178 |
| 5 | 189 | DDK | no effect | Surface residues in loop |
| 6 | 265 | KVKKK | stringent ts | K269 has structural role in the OB domain - ligase subdomain interface |
| 7 | 345 | KRK | no effect | Surface of C-terminal domain |
| 8 | 345 | KRKIK | no effect | Surface of C-terminal domain, affecting potentially secondary structure |
| ER | 185 | ER | inactive | Structural hydrogen bonds of E185 in the ligase subdomain |
| ER-5 | 185 | ERSFDDK | stringent ts | Structural hydrogen bonds of E185 in ligase subdomain |

*Mutated residues are shown in color according to their charge

**Reference**

Wang X, Ma L, Li N, Gao N. Structural insights into the assembly and mechanism of mpox virus DNA polymerase complex F8-A22-E4-H5. Mol Cell. 2023; S1097-2765(23)00909–7. doi:10.1016/j.molcel.2023.10.038
