# Supplementary material for: Structure and flexibility of the DNA polymerase holoenzyme of vaccinia virus
Source: PLoS Pathog. 2024 May 20;20(5):e1011652. doi: 10.1371/journal.ppat.1011652 (PMC11142717; doi:10.1371/journal.ppat.1011652)
Supplement: S1 Fig — (PDF) [file ppat.1011652.s004.pdf]

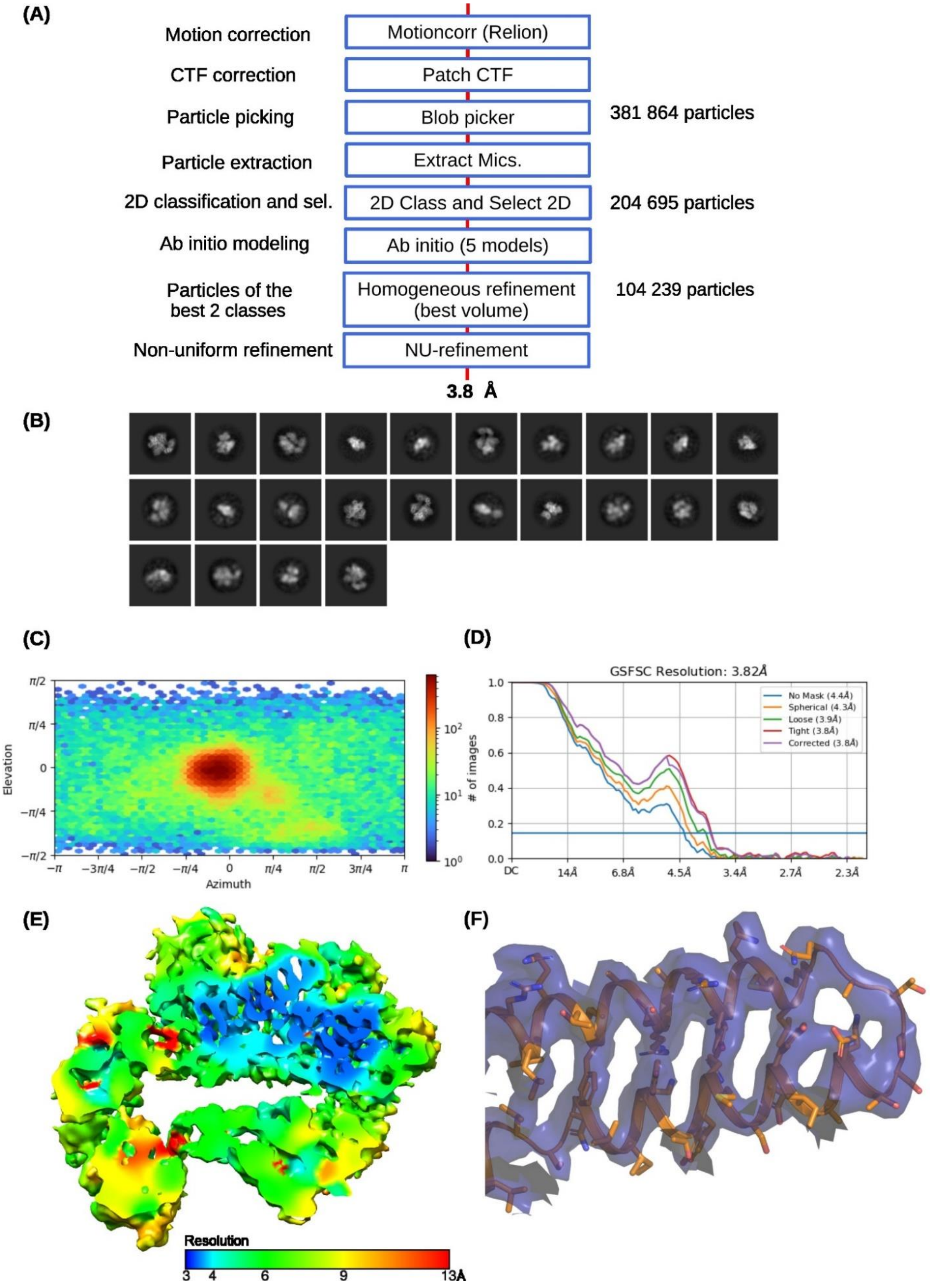

S1 Fig. Cryo-EM structure determination. (**A**) Flow-diagram of the structure determination using CryoSparc. (**B**) 2D classes containing 204 695 particles used for the 3D reconstruction. (**C**) Orientation of the particles used in the final model obtained after non-uniform refinement. (**D**) Gold standard Fourier shell correlation of the refined model. (**E**) Cut-away of the sharpened electron density map colored according to the local resolution. (**F**) Example electron density of the finger domain of E9 with the underlying structure in stick representation.
