# Supplementary material for: Structure and flexibility of the DNA polymerase holoenzyme of vaccinia virus
Source: PLoS Pathog. 2024 May 20;20(5):e1011652. doi: 10.1371/journal.ppat.1011652 (PMC11142717; doi:10.1371/journal.ppat.1011652)
Supplement: S2 Fig — (PDF) [file ppat.1011652.s005.pdf]

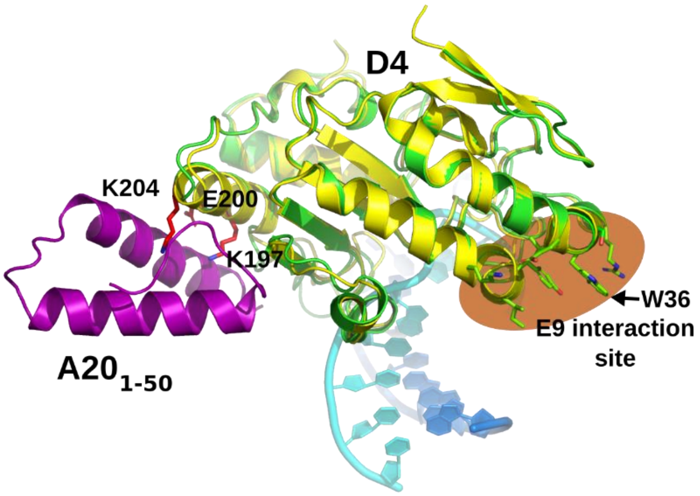

S2 Fig. The KEK mutant of VACV D4. Cartoon of the 1.3 Å crystal structure of D4KEK in green superposed onto the structure of wt D4 (yellow) in complex with the N-terminal fragment of A20 ($A20_{1\text{-}50}$ in violet) and dsDNA carrying an abasic site in blue (pdb entry 4yig). The three mutated hydrophobic residues disrupting the dimerization site of D4, which based on the structure would also interfere with the binding of A20, are indicated (F197K, I200E, L204K). The residues forming the contact with E9 in the holoenzyme structure are also shown in stick representation.
