# Supplementary material for: Structure and flexibility of the DNA polymerase holoenzyme of vaccinia virus
Source: PLoS Pathog. 2024 May 20;20(5):e1011652. doi: 10.1371/journal.ppat.1011652 (PMC11142717; doi:10.1371/journal.ppat.1011652)
Supplement: S3 Fig — (PDF) [file ppat.1011652.s006.pdf]

(A)

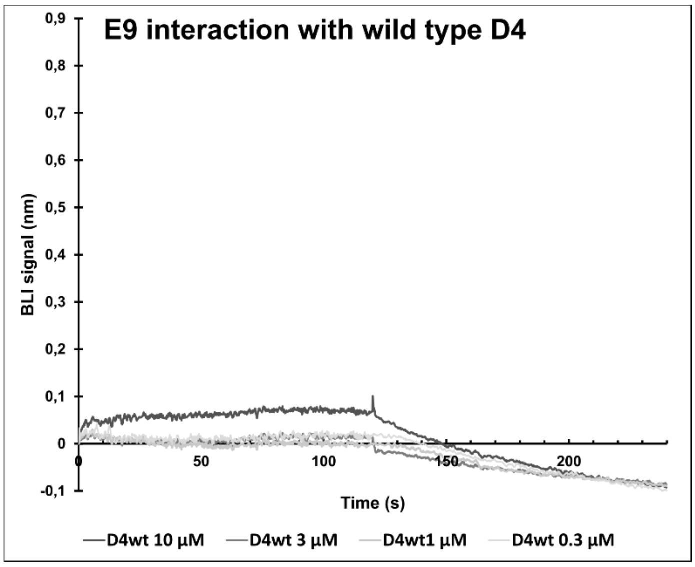

(B)

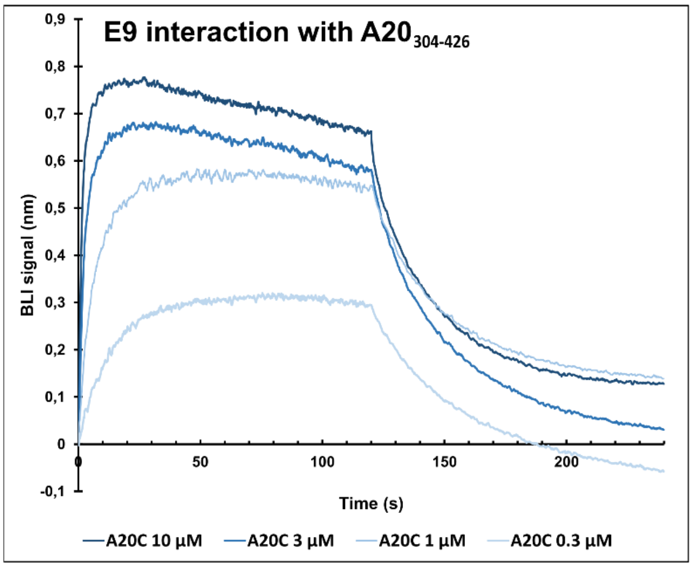

S3 Fig. Analysis of the E9 – wt D4 interaction by BLI. E9 is immobilized on a sensor tip using its 6His-tag. Data have been corrected for the unneglectable dissociation of 6His-tagged E9 from the sensor tip. (**A**) Interaction with wt D4. (**B**) Control with the interaction with $A20_{304\text{-}426}$ (A20C).
