# Supplementary material for: Structure and flexibility of the DNA polymerase holoenzyme of vaccinia virus
Source: PLoS Pathog. 2024 May 20;20(5):e1011652. doi: 10.1371/journal.ppat.1011652 (PMC11142717; doi:10.1371/journal.ppat.1011652)
Supplement: S4 Fig — (PDF) [file ppat.1011652.s007.pdf]

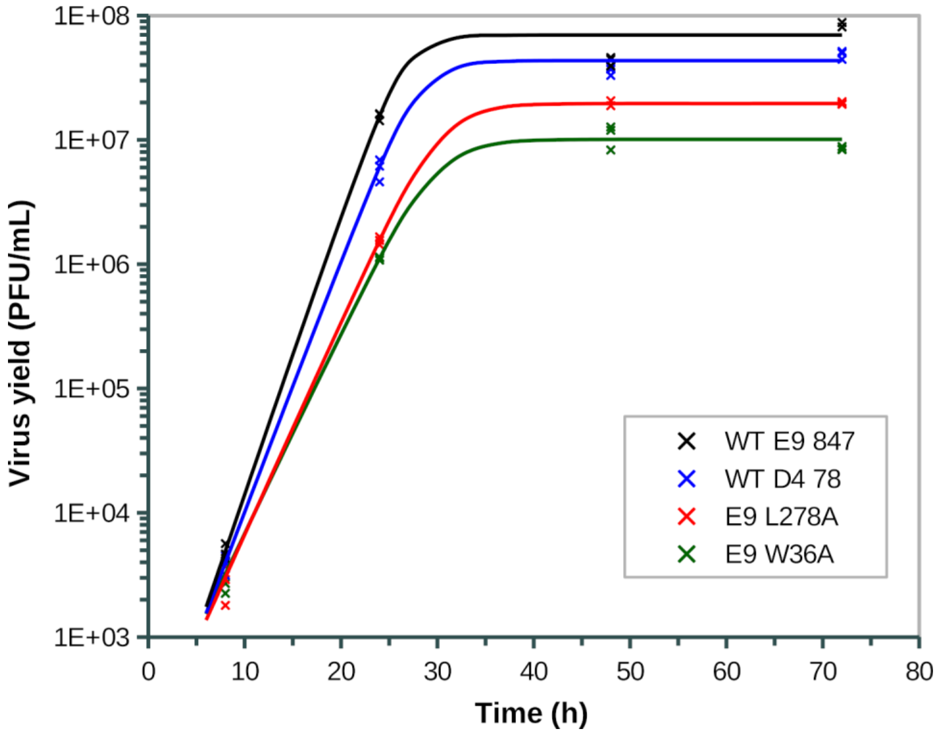

S4 Fig. Growth kinetics of VACV mutated in the E9-D4 interface. Vero cells were infected at a MOI of 0.05 and harvested at 8, 24, 48, and 72 hours post-infection. Viral titers were determined by plaque assays and expressed in PFU/mL. Data is representative of three independent experiments. Titrations were carried out in duplicate and the average is plotted. WT E9 867 and WT D4 78 are wt VACV carrying a silent mutation in *E9L* and *D4R*, respectively. E9 L278A and D4 W36A are mutant VACV with mutation at the E9-D4 interface, respectively. The data points have been fitted with logistic functions.
