# Supplementary material for: Structure and flexibility of the DNA polymerase holoenzyme of vaccinia virus
Source: PLoS Pathog. 2024 May 20;20(5):e1011652. doi: 10.1371/journal.ppat.1011652 (PMC11142717; doi:10.1371/journal.ppat.1011652)
Supplement: S5 Fig — (PDF) [file ppat.1011652.s008.pdf]

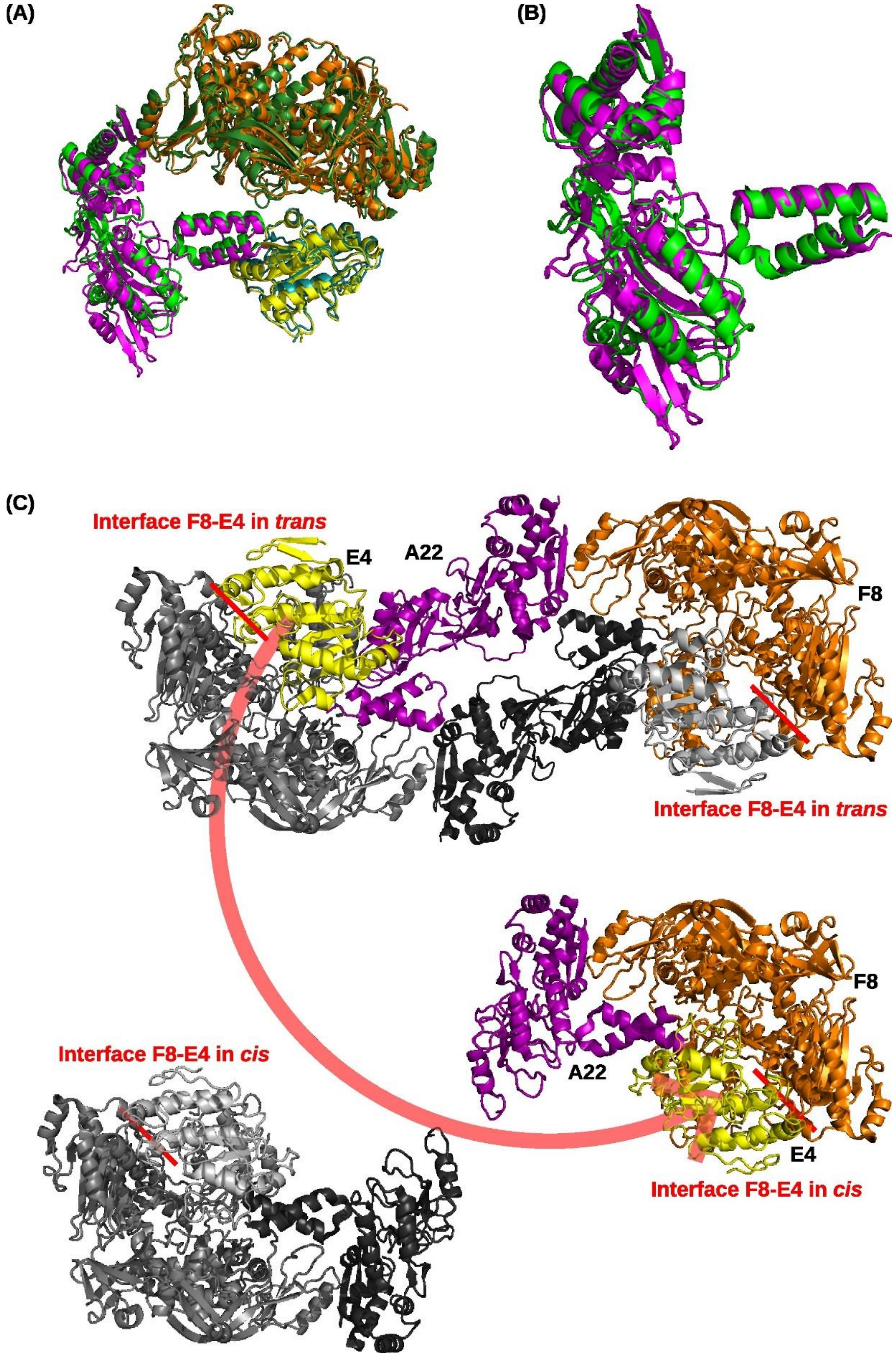

S5 Fig. Comparison of the structure of the apo form of the VACV polymerase holoenzyme with the ones of MPXV. (**A**) Superposition based on E9 and D4 of the VACV holoenzyme (orange, violet, yellow) and the MPXV one (pdb entry 8hm0, dark green, light green, cyan). (**B**) Superposition of A20 from MPXV virus (green) onto A20 of VACV (magenta). (**C**) Top: interpretation of the hexamer structure (pdb entry 8hlz) as a dimer of extended trimers. One subunit is coloured using our standard color scheme, the other one in coloured in shades of grey. The F8-E4 interface is formed in *trans*. Bottom: Formation of the compact monomer in pdb entry 8hm0 by the dissociation and rebinding of E4 forming the F8-E4 interface in *cis*. As the dimer of hexamers is specific for MPXV, exceptionally the MPXV naming of proteins has been used.
