# Supplementary material for: Structure and flexibility of the DNA polymerase holoenzyme of vaccinia virus
Source: PLoS Pathog. 2024 May 20;20(5):e1011652. doi: 10.1371/journal.ppat.1011652 (PMC11142717; doi:10.1371/journal.ppat.1011652)
Supplement: S6 Fig — (PDF) [file ppat.1011652.s009.pdf]

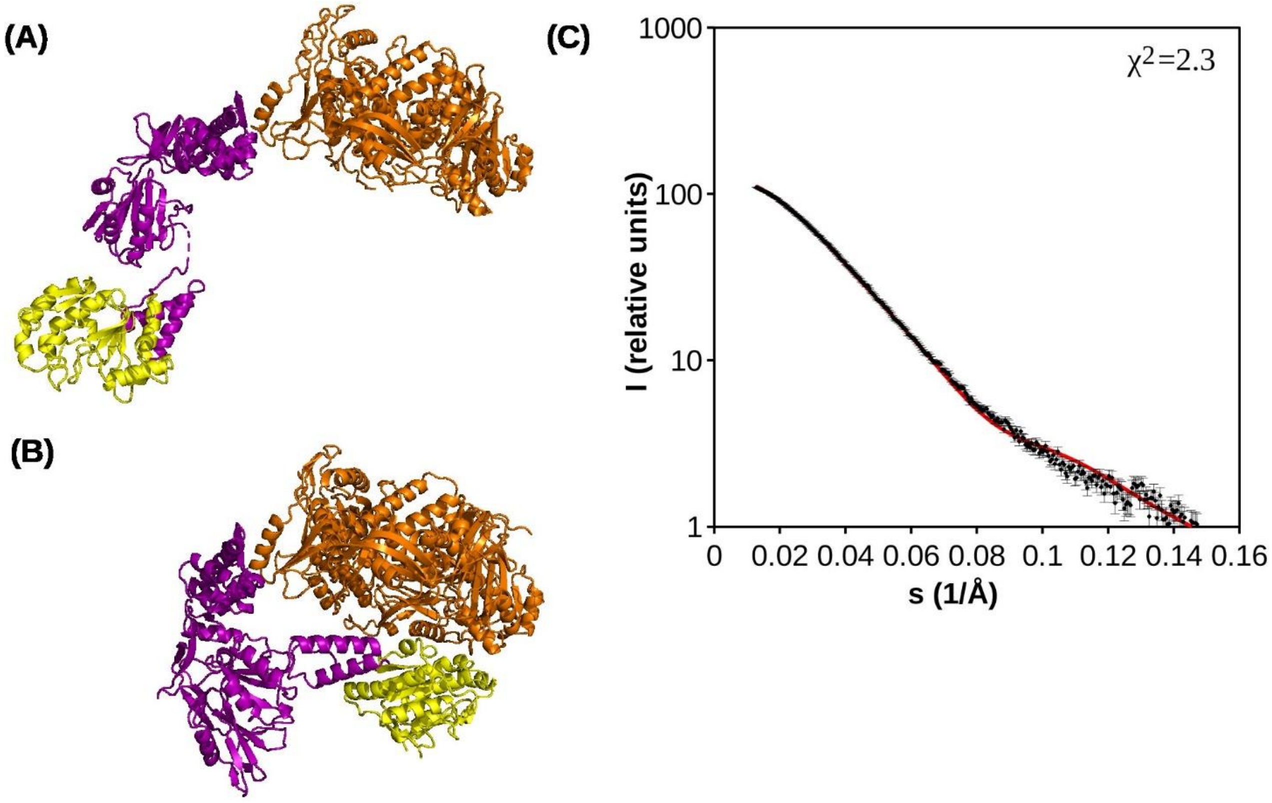

S6 Fig. Explanation of the SAXS curve of the VACV holoenzyme using 2 conformations. (**A**) Model of an extended form of the VACV heterotrimer based on pdb entry 8hlz. (**B**) Compact heterotrimer of the VACV polymerase holoenzyme obtained from cryo-EM. (**C**) SAXS curve of the VACV holoenzyme (as in Fig 3) together with a calculated curve (red) based on a mix of 58 % of the extended VACV E9-A20-D4 holoenzyme (A) and 42 % of the compact form (B).
